# Efficient Charge Carriers Separation and Transfer Driven by Interface Electric Field in FeS_2_@ZnIn_2_S_4_ Heterojunction Boost Hydrogen Evolution

**DOI:** 10.3390/molecules29174269

**Published:** 2024-09-09

**Authors:** Haijun Qiao, Rui Du, Sifan Zhou, Qi Wang, Jingyu Ren, Danjun Wang, Huifeng Li

**Affiliations:** 1 College of Science, Gansu Agricultural University, Lanzhou 730070, China; 2 College of Chemistry and Chemical Engineering, Shaanxi Key Laboratory of Chemical Reaction Engineering, Yan’an University, Yan’an 716000, China; durui199804@163.com (R.D.); zhousifan0207@163.com (S.Z.); 18391969166@163.com (Q.W.); renjingyuyau@163.com (J.R.); 3 Beijing Key Laboratory of Energy Conversion and Storage Materials, College of Chemistry, Beijing Normal University, Beijing 100875, China; lihuifeng@bnu.edu.cn

**Keywords:** FeS_2_@ZnIn_2_S_4_, S-scheme heterostructure, interface electric field, hydrogen evolution reaction, photocatalysis

## Abstract

Photocatalytic H_2_ evolution technology is regarded as a promising and green route for the urgent requirement of efficient H_2_ production. At present, low efficiency is a major bottleneck that limits the practical application of photocatalytic H_2_ evolution. The construction of high-activity photocatalysts is highly crucial for achieving advanced hydrogen generation. Herein, a new S-scheme FeS_2_@ZnIn_2_S_4_ (FeS_2_@ZIS) heterostructure as the photocatalyst was developed for enhanced photocatalytic H_2_ evolution. Density function theory (DFT) calculation results strongly demonstrated that FeS_2_@ZIS generates a giant interface electric field (IEF), thus promoting the separation efficiency of photogenerated charge carriers for efficient visible-light-driven hydrogen evolution. At optimal conditions, the H_2_ production rate of the 8%FeS_2_@ZIS is 5.3 and 3.6 times higher than that of the pure FeS_2_ and ZIS, respectively. The experimental results further indicate that the close contact between FeS_2_ and ZIS promotes the formation of the S-scheme heterojunction, where the interfacial charge transfer achieves spatial separation of charge carriers. This further broadens the light absorption range of the FeS_2_@ZIS and improves the utilization rate of photogenerated charge carriers. This work thus offers new insights that the FeS_2_-based co-catalyst can enrich the research on S-scheme heterojunction photocatalysts and improve the transfer and separation efficiency of photogenerated carriers for photocatalytic hydrogen production.

## 1. Introduction

In recent years, the conversion of solar energy into available chemical energy through artificial photosynthesis has attracted much attention. The development and utilization of renewable solar energy is expected to solve the problem of energy shortage in modern society [1,2,3]. Hydrogen energy (H_2_) is an advanced clean energy with high calorific value, no pollution, and environmentally friendly characteristics that have a broad application prospect [4,5,6]. Furthermore, according to previously reported research, the dramatic increase in global demand for hydrogen and the creation of huge revenues through the hydrogen economy have led to hydrogen energy being an important cornerstone of the plan to achieve net zero and sustainable development [7]. Photocatalytic technology can realize the conversion of solar energy to H_2_ through artificial photosynthesis, a process of pollution-free, high-energy utilization and conversion [8,9]. However, low efficiency has been a major bottleneck that limits the practical application of photocatalytic H_2_ evolution. Therefore, the design and development of new types of high-performance photocatalysts for H_2_ production are the key to solving this problem [10,11].

So far, various semiconductor materials have been found to produce H_2_ during water splitting. Among them, metal sulfide semiconductor materials, such as CdS [12,13,14], ZnS [15,16], In_2_S_3_ [17,18], etc., are widely used as H_2_ production photocatalysts because of their excellent light absorption and suitable band structure. ZnIn_2_S_4_ (ZIS) is a widely used metal sulfide photocatalyst with a wide light absorption, tunable band gap, high stability, and low toxicity [19]. However, the performance of photocatalytic H_2_ production of the pristine ZnIn_2_S_4_ is largely limited by the rapid reorganization of photoborne carriers [20,21]. Currently, many methods have been tried to improve the H_2_ production activity of single photocatalysts, such as doping, defect engineering, sensitization, and heterostructures. Generally, constructing heterostructures can effectively enhance the separation of photogenerated carriers and improve their photocatalytic properties [22,23]. Precious metals, such as Au, Pd, and Pt, have been widely recognized as effective photocatalytic co-catalysts for hydrogen production [24,25,26]. However, their low abundance and high cost have seriously restricted large-scale applications [27,28]. It is necessary to find new semiconductors as low-price alternatives to precious metals. FeS_2_ is an ideal co-catalyst with excellent electrical conductivity and light absorption ability [29,30]. At the same time, FeS_2_ and ZIS form a semiconductor–semiconductor heterojunction through the interface interaction, which is not only conducive to the rapid separation of the photogenerated carrier to accelerate HER kinetics but can also further broaden the light absorption of heterostructured catalysts [31,32,33].

Herein, a novel S-scheme FeS_2_@ZnIn_2_S_4_ (FeS_2_@ZIS) heterostructure was synthesized by a simple electrostatic self-assembly method. It was confirmed that the formation of the heterostructure broadens the light absorption range, while the S-scheme charge transfer mechanism facilitates the separation and transfer of photocarriers. The composite catalyst shows higher H_2_ evolution efficiency than the individual ZIS and FeS_2_ and also exhibits good photostability. The H_2_ production rate of the optimal composite catalyst (8% FeS_2_@ZIS) is 5.3 and 3.6 times higher than that of FeS_2_ and ZIS, respectively.

## 2. Results and Discussion

### 2.1. Structure and Morphology Analysis

**Figure 1:**
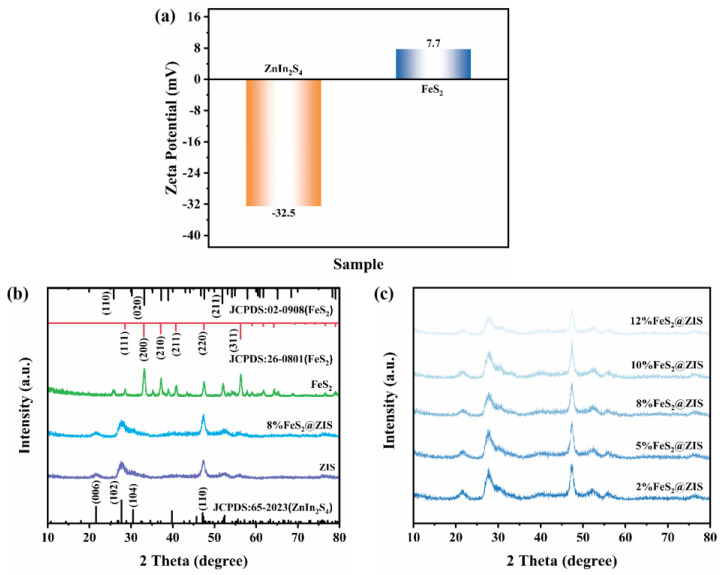
(**a**) Zeta potentials of the prepared ZnIn_2_S_4_ and FeS_2_; (**b**) XRD pattern of the prepared FeS_2_, ZnIn_2_S_4_, and FeS_2_@ZnIn_2_S_4_; (**c**) XRD pattern of x% FeS_2_@ZIS with different FeS_2_ loadings.

FeS_2_@ZIS heterostructures were prepared by the electrostatic self-assembly process. As shown in the Zeta potentiogram (Figure 1a), the pure FeS_2_ and ZIS samples have opposite electrification, 7.7 and −32.5 mV, respectively. Therefore, the FeS_2_@ZIS heterostructures are assembled by electrostatic attraction. The crystal structures of the prepared catalysts were studied by XRD. As shown in Figure 1b, four characteristic diffraction peaks appeared at 21.59°, 27.69°, 30.45° and 47.17° for the prepared ZIS, corresponding to the (006), (102), (104) and (110) crystal faces of ZIS (JCPDS Card NO. 65-2023), respectively [34]. The XRD pattern of FeS_2_ showed that it is composed of two different crystalline phases, corresponding to FeS_2_ (JCPDS Card NO. 02-0908) and FeS_2_ (JCPDS Card NO. 26-0801), respectively [35]. Due to the small content of FeS_2_, only the characteristic diffraction peak belonging to ZIS appeared in the XRD pattern of the FeS_2_@ZIS composite catalyst. Figure 1c shows the XRD patterns of FeS_2_@ZIS in different proportions. With the increase in FeS_2_ loading, the intensity of XRD diffraction peaks for the composite catalyst gradually decreased, which is because the crystallinity of the catalysts decreases upon the surface loading of FeS_2_.

**Figure 2:**
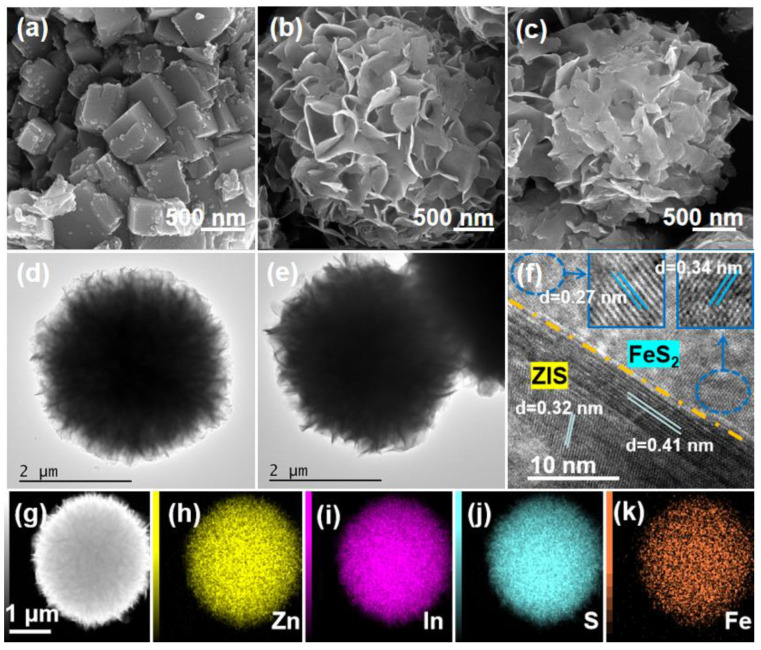
SEM images of (**a**) FeS_2_, (**b**) ZIS, and (**c**) 8%FeS_2_@ZIS; TEM images of (**d**) ZIS and (**e**) 8%FeS_2_@ZIS; (**f**) HR-TEM images of 8%FeS_2_@ZIS; and (**g**–**k**) EDX-mapping images of 8%FeS_2_@ZIS.

The structure and morphology characteristics of the prepared catalysts were analyzed by SEM and TEM tests. As shown by the SEM image in Figure 2a, pure FeS_2_ shows an irregular structure. SEM and TEM images of pure ZIS show a uniform spheroidal structure composed of a large number of flakes (Figure 2b,d). Figure 2c,e correspond to SEM and TEM images of the 8%FeS_2_@ZIS nanocomposite catalyst. Upon recombination with FeS_2_, 8% FeS_2_@ZIS also exhibits a sphere structure composed of thin nanosheets. However, the surface of the FeS_2_@ZIS complex sphere collapses due to the surface load of FeS_2_. From the HR-TEM image of the composite catalyst, the (006) and (102) crystal faces belonging to ZSI can be observed, with crystal face spacings of 0.41 nm and 0.32 nm, respectively (Figure 2f). At the same time, the (110) and (200) crystal faces belonging to FeS_2_ (JCPDS Card NO. 02-0908) and FeS_2_ (JCPDS Card NO. 26-0801) were also observed, and the crystal face spacing was 0.34 and 0.27 nm, respectively. This provided evidence for the successful preparation of the FeS_2_@ZIS composite catalyst. In addition, it can be seen from the EDX-mapping element spectrum at FeS_2_@ZIS that Zn, In, S, and Fe elements were evenly distributed in the composite sample (Figure 2g–k).

**Figure 3:**
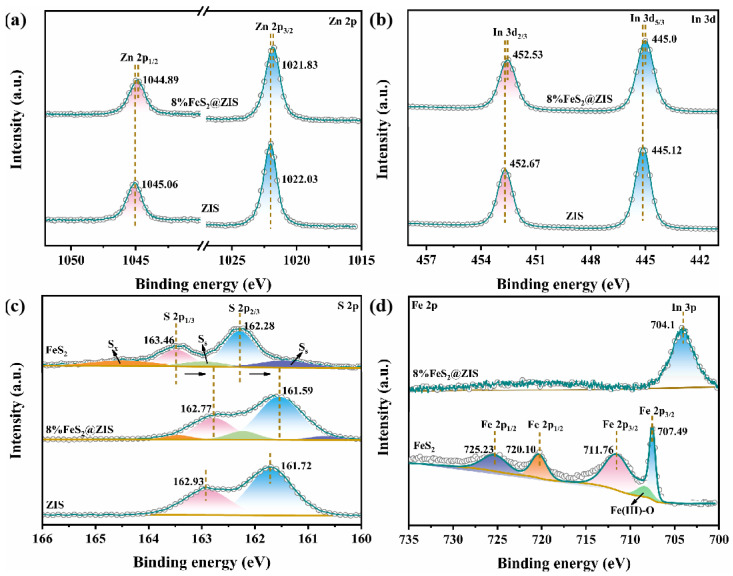
XPS spectra of the ZIS, FeS_2_, and 8%FeS_2_@ZIS heterostructure: (**a**) Zn 2p, (**b**) In 3d, (**c**) S 2p, and (**d**) Fe 2p.

The structural composition and surface valence states of the prepared catalysts were analyzed by the XPS test. The XPS survey spectrum in Figure S1 contains all the elements in ZIS and 8%FeS_2_@ZIS. In Figure 3a, the XPS high-resolution spectra of Zn 2*p* of pristine ZIS are deconvolved into two peaks, belonging to Zn 2*p*_3/2_ and Zn 2*p*_1/2_ at 1022.03 and 1045.06 eV, respectively [36]. The peaks of the binding energies at 445.12 and 452.27 eV in the In 3*d* high-resolution spectra of the ZIS correspond to In 3*d*_5/3_ and In 3*d*_2/3_, respectively (Figure 3b) [37]. As shown in Figure 3c, the S 2*p* spectrum of ZIS is deconvolved into two peaks around 161.90 and 163.10 eV, corresponding to S 2*p*_3/2_ and S 2*p*_1/2_, respectively. In the case of FeS_2_, the main S state contains five contributions: the peaks at 162.28 and 163.46 eV correspond to S 2*p*_3/2_ and S 2*p*_1/2_ of S_2_^2−^; the surfaces S_2_^2−^ at 161.46 and 162.90 eV arise from band bending in the space charge region; and S_X_ at 164.50 eV is derived from a polysulfide (S_n_^2−^) or the nuclear pore effect [38,39]. In Figure 3d, the peaks of the Fe 2*p* spectrum of FeS_2_ at 711.76 and 707.49 eV correspond to Fe 2*p*_3/2_, and the other two peaks at 725.23 and 720.10 eV correspond to Fe 2*p*_1/2_, indicating the presence of FeS_2_ [40]. Furthermore, the peak at 708.9 eV is due to the oxidation of Fe^2+^ to Fe^3+^ [41]. As shown in Figure 3a–d, after forming the FeS_2_@ZIS composite, compared with pure ZIS, the peaks of Zn 2*p*, In 3*d*, and S 2*p* in the composite shift towards the direction of higher binding energy. Conversely, the S 2*p* peak in the composite shifts towards a lower binding energy relative to FeS_2_. The opposite shift of the binding energy is caused by the interface interaction between FeS_2_ and ZIS after the formation of the heterostructure.

### 2.2. HER Performance

**Figure 4:**
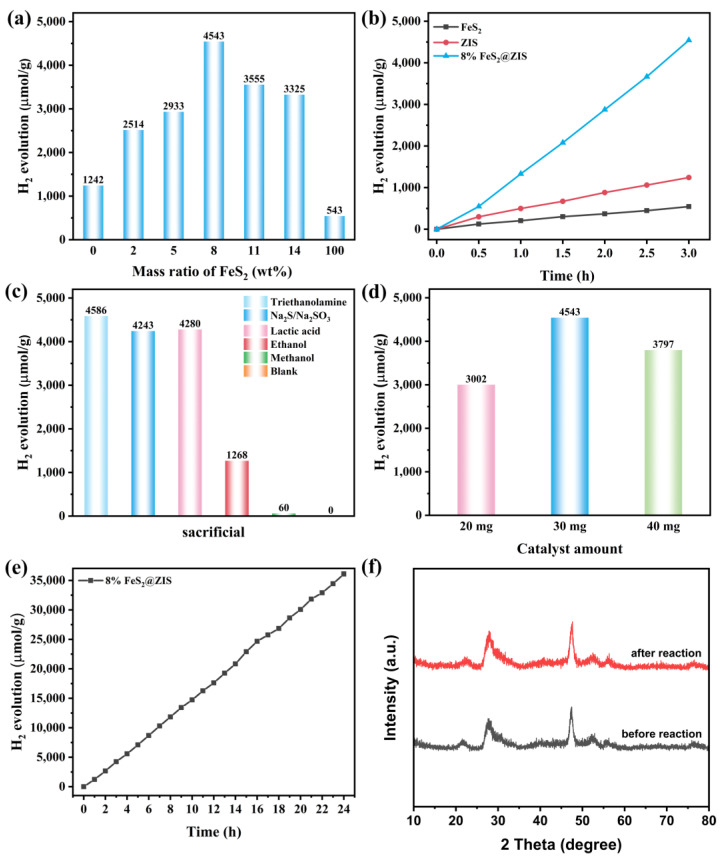
(**a**) H_2_ production activity of x% FeS_2_@ZIS for different proportions of FeS_2_ loading; (**b**) H_2_ production activity of FeS_2_, ZIS, and 8%FeS_2_@ZIS within 3 h; (**c**) Photocatalytic H_2_ production activity of 8%FeS_2_@ZIS with different sacrificial agents; (**d**) H_2_ production activity of different dosages of catalyst; (**e**) H_2_ production activity within 24 h of the 8%FeS_2_@ZIS sample; and (**f**) XRD patterns of the 8%FeS_2_@ZIS sample before and after the photocatalytic reaction.

The H_2_ production activity of the catalysts was systematically studied using Na_2_S/Na_2_SO_3_ as the sacrificial agent. Figure 4a compares the H_2_ production activity of FeS_2_@ZIS composite catalysts with different amounts of FeS_2_ loading. With the increase in FeS_2_ loading, the H_2_ production efficiency gradually increased, and the highest H_2_ production performance was obtained when the mass fraction of FeS_2_ in the composite was 8%, yielding 4543 μmol g^−1^ within 3 h. The H_2_ production rate showed a decrease when further increasing the concentration of FeS_2_ in the complex, probably because excessive FeS_2_ loading formed the carrier composite center on the surface of the catalyst, thus inhibiting the photocatalytic HER. As shown in Figure 4b, the H_2_ production performance of different catalysts within 3 h was compared, and the H_2_ production rate of the optimal composite catalyst (8%FeS_2_@ZIS) was 5.3 and 3.6 times higher than that of the pristine FeS_2_ and ZIS, respectively. Although the H_2_ production activity of 8%FeS_2_@ZIS was less active than most reports suggest, it is a significant improvement over pure FeS_2_ and ZnIn_2_S_4_ [42]. Figure 4c shows the comparison of the photocatalytic H_2_ production performance of the 8%FeS_2_@ZIS composite catalyst in different sacrificial agent systems (triethanolamine, Na_2_S/Na_2_SO_3_, lactic acid, ethanol, methanol, and without sacrificial agent). The sacrificial agent assumes the role of capturing light holes and inhibiting the carrier recombination during the photocatalytic reaction. The H_2_ production of the composite catalyst is almost zero within 3 h without a sacrificial agent, while high H_2_ production performance can be obtained in the photocatalytic system with Na_2_S/Na_2_SO_3_, triethanolamine, and lactic acid as a sacrificial agent. Figure 4d compares the effect of the catalyst dosage on H_2_ production performance. In the Na_2_S/Na_2_SO_3_ sacrifice agent system, the optimal amount of the catalyst is 30 mg, and an excessive catalyst will block the simulated sunlight in the system, thus reducing the photocatalytic H_2_ production rate. Moreover, the stability of the catalyst during the photocatalytic reactions is an important factor in the evaluation of catalyst performance. As shown in Figure 4e, the stability test of the catalyst showed that the H_2_ production of 8%FeS_2_@ZIS increased linearly within 24 h. As shown in Figure 4f and Figure S2, the XRD and SEM test results of the samples before and after the photocatalytic reaction were compared. Relative to those before the photocatalytic reaction, the crystal structure and morphology of the catalyst did not change significantly, which further proved the stability of FeS_2_@ZIS.

### 2.3. Photocatalysis Mechanism

**Figure 5:**
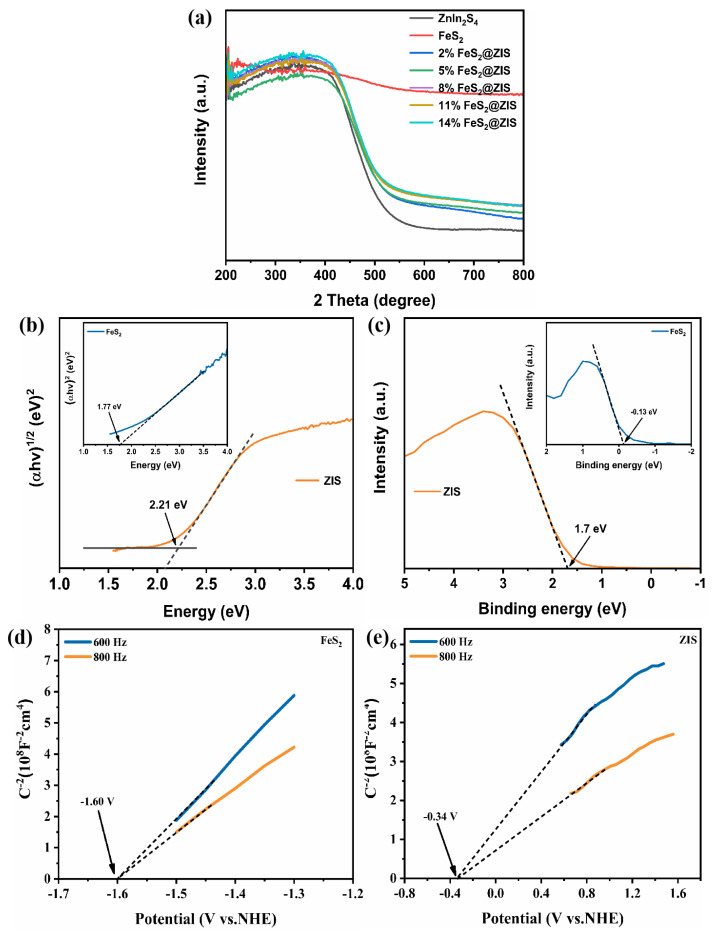
(**a**) UV-Vis DRS of FeS_2_, ZIS, and X%FeS_2_@ZIS samples; (**b**) band gap energy of FeS_2_ and ZIS; (**c**) VB-XPS spectra of FeS_2_ and ZIS; and (**d**,**e**) Mott–Schottky plot of FeS_2_ and ZIS.

The light absorption properties and band structures of the catalyst were studied by UV-Vis DRS and VB-XPS spectra. As shown in Figure 5a, the light absorption edge of the pristine ZIS was located at 530 nm, and the absorption edge of the composite sample was redshifted after the introduction of FeS_2_. With the increase in FeS_2_ concentration in FeS_2_@ZIS, the light absorption range of the composite catalyst gradually increased, and the light absorption in the visible region was enhanced. As shown in Figure 5b and the inserted figure, the energy band gaps (E_g_) corresponding to ZIS and FeS_2_ were calculated using the transformed Kubelka–Munk function and were 2.21 and 1.77 eV, respectively. The E_VBM_ of the sample was determined by the VB-XPS test, and the E_VBM_ of FeS_2_ and ZIS were −0.13 and 1.70 V, respectively (Figure 5c). In addition, for n-type semiconductors, the Fermi level (E_f_) is usually 0.1–0.3 eV below the conduction band, so the E_f_ can be obtained by the Mott–Schottky (M-S) test to further verify the band structure of the prepared catalyst [43]. As shown in Figure 5d,e, the E_f_ of FeS_2_ and ZIS were −1.60 V and −0.34 V, respectively, so the band structure of the catalyst obtained was reasonable.

**Figure 6:**
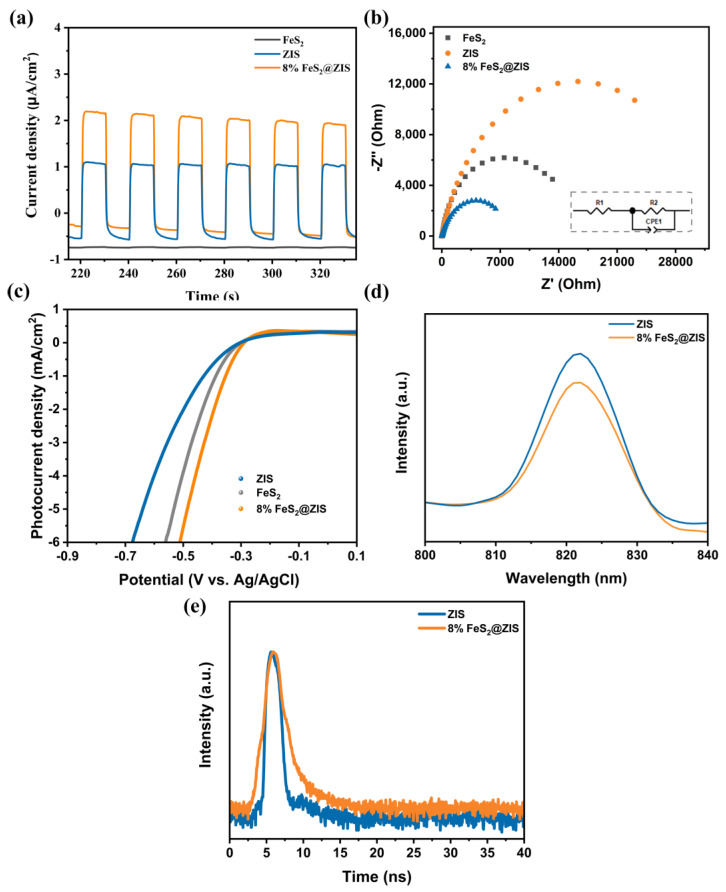
(**a**) Transient photocurrent response curves of FeS_2_, ZIS, and 8%FeS_2_@ZIS; (**b**) EIS curves of FeS_2_, ZIS and 8% FeS_2_@ZIS; (**c**) LSV curves for FeS, ZIS and 8% FeS_2_@ZIS; (**d**) PL spectra of ZIS and 8% FeS_2_@ ZIS; and (**e**) TR-PL spectra of ZIS and 8%FeS_2_@ZIS.

The photogenerated charge carrier separation properties of photocatalysts were evaluated by photoelectrochemistry, PL, and TR-PL testing. As shown by the i-t curve in Figure 6a, FeS_2_ exhibits a low photocurrent density due to the rapid photogenerated e^−^/h^+^ recombination. After the formation of the composite catalyst, 8% FeS_2_@ZIS shows enhanced photocurrent response compared with the pristine FeS_2_ and ZIS [44]. As shown in Figure 6b, the arc radius of the electrochemical impedance spectral (EIS) Nyquist plot of 8% FeS_2_@ZIS is smaller than that of bare ZIS and FeS_2_, indicating a lower interfacial charge transfer resistance [45]. The EIS spectra are composed of the ohmic resistance (R_1_), the charge transfer resistance (R_2_), and the constant phase element, where the R_2_ value can reflect the electron transport capacity of the sample [46]. Furthermore, the fitting impedance results (Figure 6b and Table S1) show that the three catalysts have a similar R_1_. Relative to the bare FeS_2_ and ZIS, the R_2_ of the 8%FeS_2_@ZIS is significantly reduced (Table S1), indicating that the charge transfer efficiency is significantly improved after the formation of the composite catalyst. The EIS result indicates that 8%FeS_2_@ZIS has the strongest separation and transfer ability of photogenerated carriers, thus promoting the photocatalyst performance. As shown in Figure 6c, the catalyst surface hydrogen production dynamics were explored by the LSV test. Compared with FeS_2_ and ZIS, the 8%FeS_2_@ZIS composite has a higher photocurrent density and a lower HER overpotential at the same potential and photocurrent density, indicating that the 8%FeS_2_@ZIS composite has the best electron transfer efficiency as an electrode [47,48]. The rapid recombination of photogenerated carriers enhances the fluorescence of catalyst material. As shown in Figure 6d, the PL intensity of 8%FeS_2_@ZIS at about 820 nm is weakened compared with the pristine ZIS, indicating that the formation of the composite effectively inhibits the photogenerated carrier recombination. The TR-PL results indicate (Figure 6e) that the average lifetime of the 8%FeS_2_@ZIS composite (2.78 ns) is increased compared to pure ZIS (2.27 ns), indicating that the heterostructure achieves more efficient charge transfer [49]. The above analyses confirm that the separation and transfer of photogenerated carriers can be accelerated through the heterogeneous interface after the formation of heterogeneous structures so as to achieve efficient carrier utilization and, thus, improve the photocatalytic hydrogen production rate.

**Figure 7:**
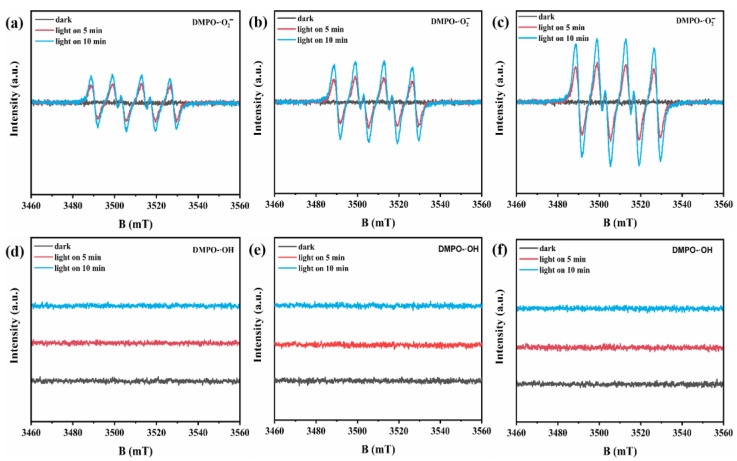
ESR spectra of DMPO-·O_2_^−^ in menthol of FeS_2_ (**a**), ZIS (**b**), and 8%FeS_2_@ZIS (**c**) under dark and light illumination; DMPO-·OH in aqueous solution of FeS_2_ (**d**), ZIS (**e**), and 8%FeS_2_@ZIS (**f**) under dark and light illumination.

The separation efficiency of photogenerated electron/hole pairs was further revealed by DMPO radical trapping experiments with ESR spectroscopy. As shown in Figure 7a–f, no radical generation was observed in all samples under dark conditions. The conduction band positions of FeS_2_ (−1.90 V) and ZIS (−0.51 V) both met the formation potential of superoxide free radicals (O_2_ + e_CB_-→·O_2_^−^ at −0.33 V vs. NHE), so four broad peaks of equal intensity were detected in the methanol solution after illumination, corresponding to the DMPO-·O_2_^−^ signal. With the extension of illumination time, the concentration of ·O_2_^−^ generated in the solution gradually increased, and thus, the peak intensity gradually increased. Since the valence band maximum of FeS_2_ (−0.13 V) and ZIS (1.77 V) did not meet the generation potential of ·OH (OH^−^ + h_VB_^+^→·OH + H^+^ at 1.99 V vs. NHE), no DMPO-·OH signal was detected under light illumination. As shown in Figure 7a–c, 8% FeS_2_@ZIS exhibited a stronger ESR signal of DMPO-·O_2_^−^ than that of both FeS_2_ and ZIS, respectively. The above analysis results show that the construction of the FeS_2_@ZIS heterojunction can effectively accelerate the transfer of photogenerated charge carriers, thus improving the photocatalytic hydrogen production performance.

To determine the effect of the specific surface area and pore structure on the hydrogen production properties, samples were tested for nitrogen adsorption–desorption. In Figure S3, ZIS and 8%FeS_2_@ZIS show similar surface areas of 68.16 m^2^ g^−1^ and 64.19 m^2^ g^−1^, respectively, and both have mesoporous structures. Therefore, the pore structure and the specific surface area of the catalyst after forming the composites did not change significantly due to the small amount of FeS_2_ loading.

**Figure 8:**
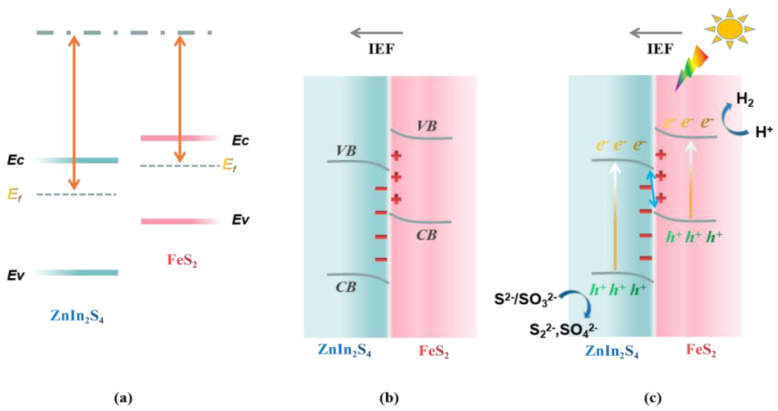
Photocatalytic mechanism for HER over the FeS_2_@ZIS S-scheme heterojunction (**a**) before contact, (**b**) upon contact, and (**c**) under illumination.

Based on the above characterization and experimental results, the charge transfer mechanism of the composite catalyst will be further discussed. As shown in Figure 8a,b, due to the staggered band structure of FeS_2_ and ZIS, spontaneous electron transfer can occur at the heterogeneous interface upon the two semiconductors being brought into contact. According to the M-S test results, the transfer direction of the electrons at the heterogeneous interface is from FeS_2_ with higher E_f_ to ZIS with lower Ef until the Fermi level of both semiconductors reaches agreement. The transfer of electrons between semiconductors leads to the formation of an internal electric field (IEF) at the heterogeneous interface that is opposite to the direction of electron flow. Due to the loss of electrons, the electric potential at the interface of FeS_2_ increases, and the band bends upward. On the contrary, the band edge at the heterojunction interface of the ZIS side is bent downward due to electron enrichment, and opposite band bending can prevent the continuous flow of electrons. As shown in Figure 8c, under light, the VB electrons of the semiconductor are excited to CB while leaving photogenerated holes in the same position of VB. Under the action of IEF, the photogenerated h^+^ of VB in FeS_2_ and the photogenerated e^-^ of CB in ZIS migrate to the heterogeneous interface and recombine. The photogenerated electrons of CB in FeS_2_ migrate to the surface and participate in the HER, while the photogenerated h^+^, retained at the VB of ZIS, is consumed by the hole-trapping agent (Na_2_S/Na_2_SO_3_). Therefore, the charge transfer of the prepared FeS_2_@ZIS heterostructures conforms to the S-scheme mechanism. 

**Figure 9:**
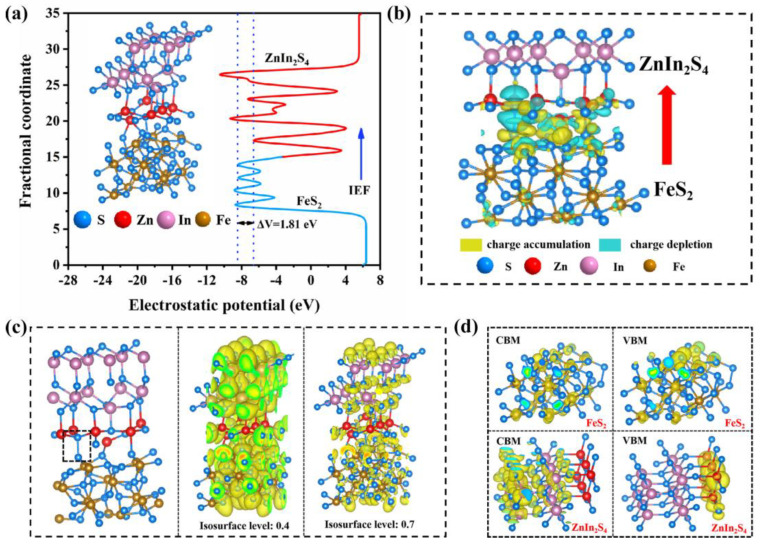
(**a**) Electrostatic potential profile of FeS_2_@ZIS; (**b**) charge density difference for FeS_2_@ZIS; (**c**) electronic local function (ELF) of FeS_2_@ZIS; and (**d**) conduction band minimum and valence band maximum charge distributions of FeS_2_ and ZnIn_2_S_4_.

The promotion of charge carrier separation by the IEF in the FeS_2_@ZIS heterostructure was further confirmed using density functional theory (DFT) calculations. At the FeS_2_/ZnIn_2_S_4_ heterogeneous interface, the electrons were expected to transfer from FeS_2_ to ZnIn_2_S_4_. The DFT results indicated that there was electron transfer potential (ΔV = 1.81 eV) between the FeS_2_ and ZnIn_2_S_4_ regions (Figure 9a), indicating the existence of an IEF directed from FeS_2_ to ZnIn_2_S_4_. The IEF drove the photogenerated electron transfer from ZnIn_2_S_4_ to FeS_2_. Figure 9b shows the charge density difference in FeS_2_@ZIS, where the yellow and blue regions represent electron accumulation and electron depletion, further confirming that the IEF from FeS_2_ to ZnIn_2_S_4_ drives the electron transfer from ZnIn_2_S_4_ to FeS_2_. 

The electronic localization function (ELF) visualizes the electron density distribution around the S-S bond (Figure 9c). The smaller the value, the stronger the delocalization of the electron, while the larger the value, the stronger the localization of the electron. When the value of the isosurface level is 0.7, no electrons appear; when the value is 0.4, electrons gather on the S-S bond. The smaller the value, the more electrons accumulate on the S-S bond, indicating strong delocalization and high electron kinetic energy. Therefore, the S-S bond acts as an electron bridge to promote charge separation, transmission, and migration. Through the distribution of charge density, it is further confirmed which atom contribute to the VBM and CBM of FeS_2_ and ZnIn_2_S_4_ (Figure 9d). The results show that the VBM and CBM of FeS_2_ are contributed by S and Fe atoms, while the VBM and CBM of ZnIn_2_S_4_ are contributed by Zn and S atoms. This further proves that holes on FeS_2_ recombine with electrons on ZnIn_2_S_4_ through the S-S bond, thus forming an S-scheme heterojunction between FeS_2_ and ZnIn_2_S_4_.

## 3. Experimental Section

### 3.1. Synthesis of FeS_2_/ZIS Photocatalyst

*Preparation of ZnIn_2_S_4_(ZIS)*. Generally, Zn(NO_3_)_2_∙6H_2_O (1 mmol) and InCl_3_∙4H_2_O (2 mmol) were dissolved in 60 mL of deionized water, and then thioacetamide (10 mmol) was added to the solution under agitation. After stirring at room temperature for 30 min, the mixture was transferred to a 100 mL hydrothermal reactor and heated at 160 °C for 12 h. The mixture is then cooled naturally to room temperature. The obtained light-yellow product was centrifuged, washed 3 times with deionized water and ethanol, and dried at 60 °C for 12 h. The resulting ZnIn_2_S_4_ is designated ZIS.

*Preparation of FeS_2_*. In total, 1 mmol of FeCl_3_·6H_2_O and 6 mmol of thiourea were dissolved in 40 mL of deionized water and stirred for 10 min. The transparent solution was then transferred to a 50 mL stainless steel autoclave coated with Teflon and reacted for 24 h at 180 °C. The reaction was cooled to room temperature, and the resulting FeS_2_ precipitate was washed by centrifugation. Finally, the products were dried at 80 °C for 24 h. 

*Preparation FeS_2_@ZIS*. The FeS_2_@ZIS photocatalyst was prepared via a facile electrostatic self-assembly method. Typically, 0.3 g of ZnIn_2_S_4_ was dissolved in 30 mL of deionized water and stirred for 30 min. Then, 0.024 g of prepared FeS_2_ was added and continuously stirred for 24 h. The obtained precipitates were washed with deionized water and dried overnight at 70 °C to obtain a FeS_2_@ZIS complex. x%FeS_2_@ZIS with a different mass ratio was prepared by changing the mass of added FeS_2_.

### 3.2. Characterization of the Samples

The crystal structure of as-prepared samples was investigated by powder X-ray diffraction (Shimadzu XRD-7000) (Kyoto, Japan). The UV-Vis diffuse reflectance spectra were used to investigate the optical characteristics of the samples (Shimadzu, UV-2550). Scanning electron microscopy (SEM, JSM-6700F) (Japan electronics, Kyoto, Japan) and transmission electron microscopy (TEM, JEOL-F200) (Japan electronics, Kyoto, Japan) were performed to observe the morphology of the samples. Raman spectroscopy was measured on a Horiba LabRAM HR Evolution instrument. X-ray photoelectron spectroscopy (XPS) was performed on a PHI-5400 (America PE) 250 xi system. Photoluminescence spectra (PL) and time-resolved photoluminescence spectra (TR-PL) were conducted on an Edinburgh FLS1000 instrument (Livingston, UK). Electron spin resonance spectra (ESR) were measured by Bruker ESR JES-FA200 (Billerica, MA, USA). 

### 3.3. Photocatalytic Activity Evaluation

The photocatalytic HER performance of the samples was carried out in a closed apparatus (Labsobar-IIIAG, Beijing Pect Light Technology Co., Ltd., Beijing, China). Typically, 30 mg of the sample was dispersed into 100 mL of Na_2_S/Na_2_SO_3_ aqueous solution. A 300 W xenon lamp was used as the light source. The produced H_2_ was tested by gas chromatography (FulliGC9790II, Taizhou, China). Comparative analysis was carried out where the Na_2_S/Na_2_SO_3_ solution was replaced with 20 mL of various sacrificial agents (methanol, ethanol, triethanolamine, and lactic acid) and 80 mL of H_2_O, while all other conditions were held constant. 

### 3.4. Theoretical Calculations

All the calculations used Vienna Ab-initio Simulation Package (VASP) to perform DFT calculations. The Perdew–Burke–Ernzerhof functional was used for exchange–correlation effects, and the DFT + D3 was employed to handle weak interactions. The cut-off energy for the plane-wave basis was 450 eV. K-points were 2 × 2 × 1 in the Brillouin zone. A 15 Å layer vacuum was applied at Z-axis of slab models to avoid the periodic effect. Energy and maximum stress were converged to 10^−5^ eV and 0.03 eV/Å, respectively.

## 4. Conclusions

Novel FeS_2_@ZIS nanocomposites were constructed by an electrostatic self-assembly approach for photocatalytic H_2_ evolution, and the obtained FeS_2_@ZIS photocatalyst exhibited highly enhanced H_2_ evolution. Morphology, optical, and textural properties and the phase structure of the heterojunction of FeS_2_@ZIS nanocomposites were investigated in detail. The obtained heterostructures showed the following advantages over pristine FeS_2_ and ZIS: firstly, the S-scheme charge transfer mechanism promotes photogenerated carrier separation and transfer and improves the utilization of carriers through interfacial charge transfer; secondly, the formation of the heterostructure broadens the light absorption range of the nanocomposite catalyst, and improves the utilization rate of light energy; thirdly, the H_2_ production rate of the optimal composite catalyst (8%FeS_2_@ZIS) is 5.3 and 3.6 times that of pristine FeS_2_ and ZIS, respectively; and, fourthly, the stability experiments show that the FeS_2_@ZIS sample can maintain a stable rate of H_2_ generation with good photocatalytic durability. Therefore, this work provides an effective way to fabricate new types of heterostructure-based photocatalysts for efficient H_2_ evolution for promising clean energy applications.

## Data Availability

Data are available on request due to restrictions, e.g., privacy or ethical.

## Supplementary Materials

The following supporting information can be downloaded at https://www.mdpi.com/article/10.3390/molecules29174269/s1: Figure S1: XPS survey spectra of ZIS and 8%FeS_2_@ZIS heterostructure; Figure S2: SEM of FeS_2_@ZIS after photocatalytic reaction; Figure S3: N_2_ adsorption–desorption isotherms (a) and pore size distribution curves (inset) of ZIS and FeS_2_@ZIS (b); Table S1: The equivalent circuit model for EIS of samples. Table S2: The comparison of H_2_ evolution rate over FeS2@ZnIn2S4 photocatalysts with previously published results [50,51,52,53,54,55,56,57,58].
