# Supplementary material for: Efficient Charge Carriers Separation and Transfer Driven by Interface Electric Field in FeS2@ZnIn2S4 Heterojunction Boost Hydrogen Evolution
Source: Molecules. 2024 Sep 9;29(17):4269. doi: 10.3390/molecules29174269 (PMC11396988; doi:10.3390/molecules29174269)
Supplement: Supplementary file 1 [file molecules-29-04269-s001.zip › molecules-3185689-supplementary.pdf]

Supporting Information

FeS_2@$ZnIn_2S_4$ S-Scheme Photocatalyst for Enhanced Photocatalytic H_2 Evolution

Haijun Qiao [1,*], Rui Du [2], Sifan Zhou [2], Qi Wang [2], Jingyu Ren [2], Danjun Wang [2,*] and Huifeng Li [3]

[1] College of Science, Gansu Agricultural University, Lanzhou 730070, China
[2] College of Chemistry and Chemical Engineering, Shaanxi Key Laboratory of Chemical Reaction Engineering, Yan'an University, Yan'an 716000, China
[3] Beijing Key Laboratory of Energy Conversion and Storage Materials, College of Chemistry, Beijing Normal University, Beijing 100875, China
* Correspondence: qiaohj_199909@163.com (H.Q.); wangdj761118@163.com (D.W.)

CONTENTS

METHODS

Figure S1 XPS survey spectra of ZIS and 8%FeS_2@ZIS heterostructure.

Figure S2 SEM after the photocatalytic reaction of the FeS_2@FeS_2@ZIS sample.

Figure S3 (a) N_2 adsorption-desorption isotherms and (b) pore size distribution curves (inset) of ZIS and FeS_2@ZIS

Table S1 The equivalent circuit model for EIS of samples.

Table S2. The comparison of H_2 evolution rate over FeS_2@$ZnIn_2S_4$ photocatalysts with previously published results.

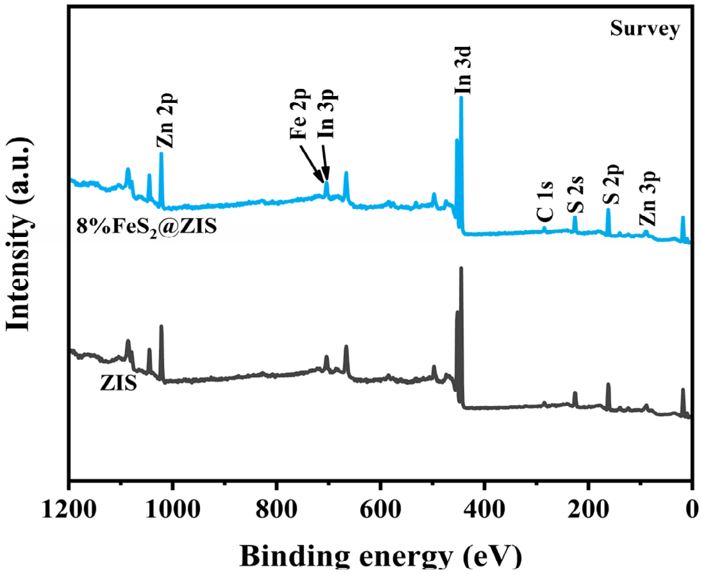

Figure S1 XPS survey spectra of ZIS and 8%FeS_2@ZIS heterostructure.

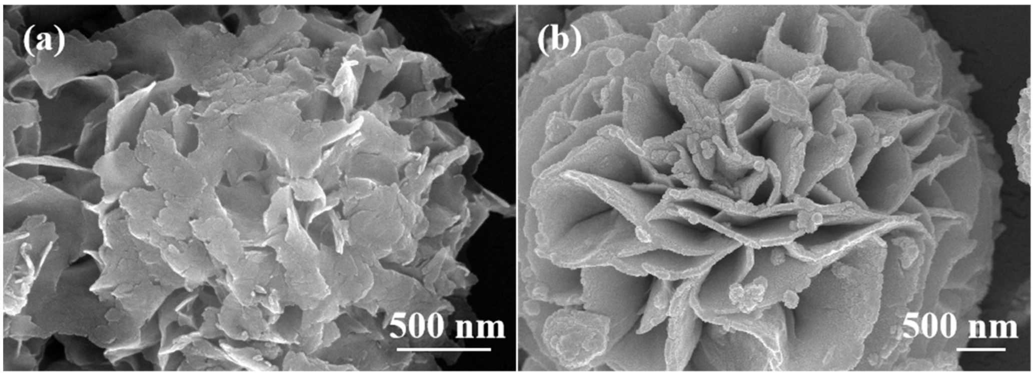

Figure S2 SEM after the photocatalytic reaction of the FeS_2@ZIS sample.

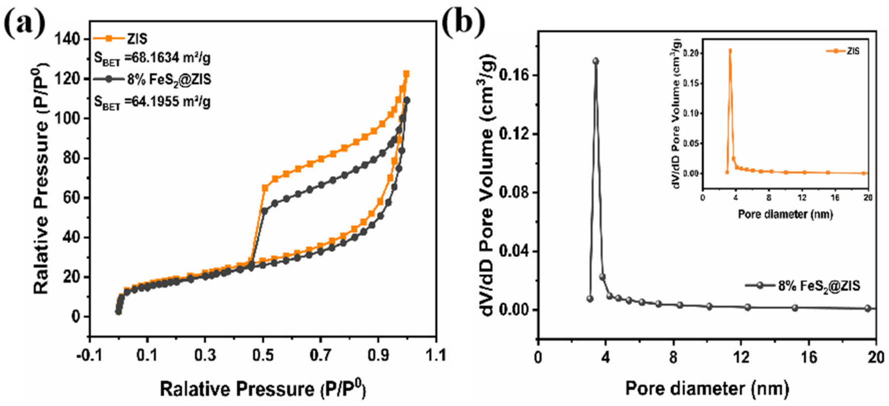

Figure S3 (a) N_2 adsorption-desorption isotherms and (b) pore size distribution curves (inset) of ZIS and FeS_2@ZIS.

Table S1 The equivalent circuit model for EIS of samples.

Samples	FeS_2	$ZnIn_2S_4$	8%FeS_2@ZIS
R_1/Ω	10.28	6.829	5.268
R_2/Ω	18582	32366	8230

Table S2. The comparison of H_2 evolution rate over FeS_2@$ZnIn_2S_4$ photocatalysts with previously published results.

Photo-catalysts	Light source	Activity (μmol g^{-1} h^{-1})	Ref.
ZnO/$CuInS_2$	300W Xe lamp	698.04	1
PtSA/Cs_2SnI_6	300W Xe lamp	430	2
CoS_2/$MgIn_2S_4$	300W Xe lamp	290	3
FeS_2/CdS	300W Xe lamp	154	4
Bi_2O_2Se/CdS	300W Xe lamp	425	5
Pt/CdS/SiC/TiO_2	300W Xe lamp	1090.4	6
Y-CeO_2/$ZnIn_2S_4$	300W Xe lamp	857	7
$CuInS_2$/$ZnIn_2S_4$	300W Xe lamp	284.9	8
TiO_2/$ZnIn_2S_4$	300W Xe lamp	348.21	9
FeS_2@$ZnIn_2S_4$	300W Xe lamp	1514.3	Present work

Reference

1. Qiao, F.; Liu, W.; Yang, J.; Liu, Y.; Yuan, J. Fabrication of ZnO/$CuInS_2$ Heterojunction for Boosting Photocatalytic Hydrogen Production. *Int. J. Hydrogen Energy*, **2024**, *53*, 840-847.

2. Zhou, P.; Chen, H.; Chao, Y.; Zhang, Q.; Zhang, W.; Lv, F.; Guo, S. Single-Atom Pt-I_3 Sites on All-Inorganic Cs_2SnI_6 Perovskite for Efficient Photocatalytic Hydrogen Production. *Nat. Commun.*, **2021**, *12*, 4412.

3. Li, J.; Yao, J.; Yu, Q.; Zhang, X.; Carabineiro, S. A.; Xiong, X.; Lv, K. Understanding the Unique Ohmic-Junction for Enhancing the Photocatalytic Activity of CoS_2/$MgIn_2S_4$ towards Hydrogen Production. *Appl. Catal. B*, **2024**, *351*, 123950.

4. Yao, B.; Wang, S.; Yao, H.; Pang, X.; Li, Y.; Sun, J. Photothermal and Pyroelectric Effects in Hollow FeS_2/CdS Nanocomposites for Enhanced Photocatalytic Hydrogen Evolution. *Surf. Interfaces*, **2024**, *49*, 104370.

5. Liu, J.; Qiu, L.; Liu, Z.; Tang, Y.; Cheng, L.; Chen, Z.; Duo, S. Boosting the Photocatalytic Activity for H_2 Production of Bi_2O_2Se/CdS Heterojunction. *Mater. Lett.*, **2023**, *345*, 134498.

6. Nagakawa, H.; Ochiai, T.; Takekuma, Y.; Konuma, S.; Nagata, M. Effective Photocatalytic Hydrogen Evolution by Cascadal Carrier Transfer in the Reverse Direction. *ACS omega*, **2018**, *3*,

12770-12777.
7. Lu, X.; Quan, L.; Hou, H.; Qian, J.; Liu, Z.; Zhang, Q. Fabrication of 1D/2D Y-doped $CeO_2/ZnIn_2S_4$ S-Scheme Photocatalyst for Enhanced Photocatalytic H_2 Evolution. *J. Alloy. Compd.*, **2022**, *925*, 166552.
8. Li, F.; Liao, B.; Shen, J.; Ke, J.; Zhang, R.; Wang, Y.; Niu, Y. Enhancing Photocatalytic Activities for Sustainable Hydrogen Evolution on Structurally Matched $CuInS_2/ZnIn_2S_4$ Heterojunctions. *Molecules*, **2024**, *29*, 2447.
9. Wei, N.; Wu, Y.; Wang, M.; Sun, W.; Li, Z.; Ding, L.; Cui, H. Construction of Noble-Metal-Free TiO_2 Nanobelt/$ZnIn_2S_4$ Nanosheet Heterojunction Nanocomposite for Highly Efficient Photocatalytic Hydrogen Evolution. *Nanotechnology*, **2018**, *30*, 045701.
